# Inter-laboratory variability of caspofungin MICs for *Nakaseomyces glabrata* isolates – an Irish tertiary hospital experience

**DOI:** 10.1099/acmi.0.000617.v4

**Published:** 2023-10-09

**Authors:** Saied Ali, Meadhbh Collison, Sinead McNicholas, Sinead McDermott

**Affiliations:** ^1^​ Department of Clinical Microbiology, St. Vincent’s University Hospital, Elm Park, Dublin 4, Ireland

**Keywords:** antifungal, antimicrobial susceptibility testing, caspofungin, echinocandin, *Nakaseomyces glabrata*

## Abstract

**Background.:**

*Nakaseomyces glabrata*, formerly *Candida glabrata*, is an opportunistic yeast and emerging cause of human infections. The use of broth microdilution (BMD) methodologies for caspofungin (CSP) antifungal susceptibility testing (AFST) against *N. glabrata* is reported to be prone to high inter-laboratory variation. We aimed to compare CSP MICs of *N. glabrata* isolates from our institution with those obtained by the Reference Laboratory for the same isolates.

**Methods.:**

All clinically significant *N. glabrata* isolates from 2019 to 2021 inclusive were reviewed. AFST was performed locally using the VITEK2 system with the AST-YS08 card, while E-tests were performed at the Mycology Reference Laboratory (MRL), and agreement between these two methods was evaluated – categorical and essential.

**Results.:**

Forty-one isolates were reviewed during the study period – 30 from blood cultures, seven from intra-operative theatre specimens and four from sterile site drain fluids. Despite an essential agreement of 100 % within ±2 log_2_ dilutions, marked discrepancies were noted in interpretative breakpoints between assays with 17 Minor and 16 Major category errors. Categorical agreement was 19.5 %, with the VITEK2 over-estimating resistance. A Mann–Whitney U-test assessed the relationship of MICs across the AFST modalities, and a statistically significant difference was noted, *P*<0.01, with a higher mean rank for VITKEK2 outputs.

**Conclusion.:**

While the VITEK2 system is highly applicable, its performance for CSP AFST is unreliable and potentially results in the mis-classification of susceptible isolates as highlighted in our study. The use of VITEK2 AST-YS08 micafungin as a sentinel echinocandin should be explored and/or the evaluation of CSP-specific E-tests as utilized by the MRL. These methods appear more consistent and less prone to the variation seen with BMD for CSP.

## Data Summary

The authors confirm all supporting data, code and protocols have been provided within the article.

## Introduction

*Candida* species are the most common cause of superficial and invasive opportunistic mycoses, representing the fourth most frequently isolated bloodstream pathogen in the USA [1–3]. Healthcare advances in immunomodulatory therapy, antimicrobial consumption, and increasing utilization of implantable and prosthetic devices have contributed to the steady increase of *Candida* infections resulting in a marked increase in morbidity and mortality among at-risk patients with significant economic costs [1, 2].

Contemporaneous surveillance data from the Centers for Disease Control and Prevention (CDC) reported an all-cause mortality rate of 25 % among persons with candidaemia [4], while the National Epidemiology of Mycoses Survey (NEMIS) in the UK reported up to 41 % for those in Intensive Care [5]. Estimated mean hospitalization, diagnostic and therapeutic costs per patient with invasive candidiasis range from $48 487 to $157 574 globally, with discrepancies between Western and Eastern Hemispheres due to availability of resources [6, 7].

According to the Prospective Antifungal Therapy (PATH) Alliance Database and the ARTEMIS DISK Global Antifungal Surveillance study, *Candida albicans* tends to be the predominant causative pathogen with a prevalence as high as 54.4 % in the USA and 62 % worldwide [8, 9]; however, its prevalence has been steadily decreasing with a concomitant increase in non-*albicans Candida* species – namely *C. glabrata*, now known as *Nakaseomyces glabrata*, in northern Europe and the USA where it is currently the second most-commonly isolated bloodstream *Candida* species and accounts for up to 46.4 % of all *Candida* infections [10, 11].

Amphotericin B and azole antifungals had been the backbone of antifungal therapy, but with the high incidence of infusion-related toxicity, nephrotoxicity and high financial costs with amphotericin B, and the emergence of fluconazole non-susceptible strains, echinocandins were developed [12]. They are well-tolerated and cost-effective and act by non-competitively inhibiting β-1,3 glucan synthase, a key enzyme necessary for maintaining the integrity of the fungal cell wall [13]. They are commonly recommended as first-line antifungals for the treatment of invasive candidiasis, as is the case at our institution, with caspofungin (CSP) being our echinocandin of choice. As such, the increasing incidence of *N. glabrata* is of particular concern due to its ability to acquire resistance to echinocandins through mutations in the FKS1 and/or FKS2 gene which encodes β-1,3 glucan synthase [12]. Early diagnosis and effective management with targeted antifungal therapy are vital for favourable patient outcomes, and necessitate reliable *in vitro* antifungal susceptibility testing (AFST).

Broth microdilution (BMD) as recommended by the Clinical and Laboratory Standards Institute (CLSI) and European Committee for Antimicrobial Susceptibility Testing (EUCAST) is considered the gold-standard for AFST for *Candida* species [14–18]. While standardized, it promotes consistency and facilitates comparison of results between different laboratories. However, its application in routine laboratories, like ours, is greatly limited as processes are tedious, time-consuming and labour-intensive requiring experienced laboratory personnel and significant resource investment [18]. Consequently, various commercial systems have been developed, including the inexpensive disc diffusion and gradient diffusion-based E-test (bioMérieux), the colorimetric Sensititer YeastOne (Trek Diagnostic Systems) and the VITEK2 (bioMérieux) which is currently employed locally [18]. VITEK2 is a fully automated BMD system which spectrophotometrically evaluates microbial growth and assesses susceptibility using various cards containing dilutional wells of specific antimicrobial agents [19, 20].

However, the use of automated BMD systems for AFST with CSP has been reported to be prone to high inter-laboratory variation [21–23]. The exact mechanism for this variation has yet to be fully elucidated but theories postulate a possible ‘eagle effect’ [24]. bioMérieux has even issued a limitation for the evaluation of CSP MICs for *N. glabrata*. Non-susceptible strains were unavailable for comparative testing during the validation of the VITEK2 system for AFST and therefore isolates yielding non-susceptible breakpoints should be submitted to a Reference Laboratory and MICs not reported without this confirmatory testing. Additionally, the Food and Drug Administration (FDA) has withdrawn its recommendation for CSP VITEK2 AFST for *N. glabrata* due to inconsistencies in MICs [25, 26].

It was observed locally that CSP VITEK2 MICs for *N. glabrata* were rarely susceptible based on clinical breakpoints, raising concerns over increasing antifungal resistance and possible therapeutic failures. Antifungal therapy was often escalated to amphotericin B while awaiting confirmatory AFST evaluation by the Reference Laboratory, which frequently returned susceptible by their testing modality. Our present study aimed to retrospectively compare CSP MICs obtained locally to those obtained by the Reference Laboratory for the same *N. glabrata* isolates among our patient cohort from 2019 to 2021 inclusive as a means to better understand the reliability of the VITEK2 for CSP AFST. AFST was performed using the VITEK2 system with the AST-YS08 card in-house while E-tests were performed at the Reference Laboratory, and agreement between these two methods was evaluated.

## Methods

All clinically significant *N. glabrata* isolates from blood cultures, bodily drain fluids from presumed sterile sites and intra-operative theatre samples with CSP AFST performed at our University Hospital laboratory from 2019 to 2021 inclusive were reviewed. Isolates were identified to the genus and species level using the bioMérieux VITEK-MS matrix-assisted laser desorption/ionization-time of flight (MALDI-TOF) instrument. CSP MICs were obtained locally using the automated VITEK2 system with the AST-YS08 card as per the manufacturer’s instructions and all strains were referred to the Mycology Reference Laboratory (MRL), Southmead Hospital, Bristol, UK, for confirmatory CSP AFST testing via E-tests [18–20, 27]. Results from the MRL are communicated electronically and via regular post, usually within 2 weeks of dispatching the isolate of reference. All MICs were interpreted as per CLSI breakpoints and they were compared using categorical and essential agreement [28], as well as a Mann–Whitney U-test. An MIC≤0.12 µg ml^−1^ was classified as susceptible, 0.25 µg ml^−1^ as intermediate and ≥0.5 µg ml^−1^ as resistant [15].

Categorical agreement (CA) is based on interpretive breakpoints – with discrepancies between the reference method (MRL) and alternative method (VITEK2) reported as Minor, Major or Very Major Errors [28]. Minor Errors (MIEs) are differences of susceptible versus intermediate or intermediate versus resistant for a given isolate. Major Errors (MEs) are resistant results by the alternate method and susceptible results by the reference method. A Very Major Error (VME) is a susceptible result by the alternative method and a resistant result by the reference method.

Essential agreement (EA) or doubling dilution difference in the MIC was calculated as the difference between log_2_(MRL MIC) and log_2_(VITEK MIC), whereby the agreement of the two methods within ±2 is the EA [18, 29].

## Results

Forty-one (41) clinically significant strains of *N. glabrata* were reviewed during the study period. Thirty isolates were obtained from blood cultures, seven (7) from intra-operative theatre specimens and four (4) from presumed sterile site drain fluids. MIC distribution with corresponding interpretation as per current breakpoints between testing platforms are shown in Table 1. A Mann–Whitney U-test was performed to assess the relationship of MICs across the AFST modalities, and a statistically significant difference was found, *P*<0.01, with a higher mean rank for VITKEK2 outputs.

**Table 1.** Sources of *N. glabrata* isolates with corresponding MICs between AFST platforms

Isolate	Source	MIC (µg ml^–1^)		Interpretation	
		Local – VITEK2	MRL – E-test	Local – VITEK2	MRL – E-test
1	Blood culture	0.5	0.25	R	I
2	Blood culture	0.25	0.125	I	S
3	Blood culture	0.5	0.125	R	S
4	Blood culture	0.25	0.125	I	S
5	Blood culture	0.5	0.06	R	S
6	Blood culture	0.5	0.125	R	S
7	Blood culture	0.5	0.125	R	S
8	Blood culture	0.5	0.125	R	S
9	Drain fluid	0.5	0.06	R	S
10	Blood culture	0.25	0.125	I	S
11	Theatre specimen	0.25	0.125	I	S
12	Blood culture	0.25	0.125	I	S
13	Theatre specimen	0.25	0.125	I	S
14	Blood culture	0.25	0.06	I	S
15	Theatre specimen	0.25	0.125	I	S
16	Blood culture	0.25	0.125	I	S
17	Theatre specimen	0.25	0.25	I	I
18	Blood culture	0.25	0.125	I	S
19	Blood culture	<0.12	0.06	S	S
20	Blood culture	0.5	0.125	R	S
21	Drain fluid	0.25	0.125	I	S
22	Blood culture	0.25	0.125	I	S
23	Blood culture	0.25	0.125	I	S
24	Theatre specimen	0.5	0.125	R	S
25	Blood culture	0.5	0.125	R	S
26	Blood culture	0.5	0.125	R	S
27	Blood culture	0.25	0.25	I	I
28	Blood culture	0.25	0.125	I	S
29	Blood culture	0.5	0.125	R	S
30	Blood culture	0.5	0.125	R	S
31	Blood culture	0.5	0.125	R	S
32	Theatre specimen	<0.12	0.125	S	S
33	Blood culture	0.5	0.125	R	S
34	Theatre specimen	<0.12	0.125	S	S
35	Blood culture	<0.12	0.125	S	S
36	Blood culture	0.5	0.06	R	S
37	Blood culture	0.25	0.5	I	R
38	Blood culture	<0.12	0.125	S	S
39	Drain fluid	0.5	0.125	R	S
40	Drain fluid	<0.12	0.125	S	S
41	Blood culture	<0.12	0.25	S	I

I, intermediate; R, resistant; S, susceptible.

**Table 2.** Categorical agreement with category errors between AFST platforms

Error type	VITEK2 result	MRL result	No. of isolates	Total no. of isolates
Complete agreement	Susceptible	Susceptible	6	
	Intermediate	Intermediate	2	8
	Resistant	Resistant	0	
Minor	Susceptible	Intermediate	1	
	Intermediate	Susceptible	14	17
	Intermediate	Resistant	1	
	Resistant	Intermediate	1	
Major	Resistant	Susceptible	16	16
Very Major	Susceptible	Resistant	0	0

Marked discrepancies were noted between interpretative breakpoints between assays as shown in Table 1, producing 33 Category Errors – 17 MIE, 16 ME and 0 VME. Complete agreement was noted for 9 isolates producing a CA of 19.5%, with the VITEK2 over-estimating resistance as shown in Tables 1 and 2.

A look at doubling dilution differences in Table 3 revealed an EA of 100% within ±2 log_2_ dilutions.

**Table 3.** Distribution of differences in MICs between AFST platforms

No. of isolates with an MIC difference of:							Agreement within ±2 log_2_ dilution, *n* (%)
< −2	2	1	0	+1	+2	> +2	
0	16	15	8	2	0	0	41 (100)

## Discussion

With increasing antifungal resistance among *Candida* species, widespread use of AFST in clinical laboratories is warranted to allow effective management, treatment and surveillance monitoring of these critical infections. The cumbersome and resource-intensive BMD method endorsed by the CLSI and EUCAST is difficult to implement in routine laboratories. Commercial automated systems such as the VITEK2 are more attractive with their streamlined set-up, automated reading and interpretation, rapid results and generation of less biohazardous waste [18].

In many countries, including the USA and Republic of Ireland, the prevalence of *N. glabrata* is increasing [1–3]. A recent clinical audit conducted locally at our hospital in the Republic of Ireland revealed that *N. glabrata* accounted for 40 % of all cases of candidaemia over the preceding year, with a further number isolated in significant clinical specimens, including bodily drain fluids from presumed sterile sites and intra-operative theatre samples [30].

At our institution, echinocandins are recommended as the first-line antifungals for treatment of invasive candidiasis. These are acylated cyclic hexapeptides which disrupt the stability of the fungal cell wall; examples include CSP, anidulafungin (AFG) and micafungin (MYC) [13]. CSP was the first drug in this class to be approved by both the FDA and the European Medicines Agency (EMA) and is indicated for oesophageal candidiasis, invasive candidiasis including candidaemia, empirical therapy in febrile neutropenia and salvage therapy for invasive aspergillosis [31]. Additionally, Bruynesteyn *et al*. conducted a cost-effectiveness analysis using a decision-tree model of CSP versus liposomal amphotericin B, and found that CSP was not only a cost-effective therapy, but may also generate savings in treatment costs and gains in quality-adjusted life years when taking into account adverse drug events [32]. As such, it became our empiric echinocandin.

However, it has been reported that the aforementioned BMD methodologies for CSP AFST are prone to unacceptably high inter-laboratory variation, and potentially result in the mis-classification of susceptible isolates, as clearly evidenced in our study [22].

This variability is not well understood but may be in part due to well-described ‘eagle effects’ among *Candida* species. When echinocandin AFST is performed under standardized conditions, an unexpected paradoxical effect may be observed where certain strains may be susceptible to low concentrations of echinocandin but may proliferate at higher concentrations [24]. Inconsistencies in the potency and stability of differing lots and variations in storage conditions have all be proposed as potential confounders [22].

The CLSI recommends that if an isolate tests susceptible to CSP, it can be safely reported as susceptible, but intermediate or resistant strains should be confirmed through alternative echinocandin AFST with either AFG or MYC [16]. Similarly, the information leaflet for AST-YS08 cards used for VITEK2 AFST reiterates this recommendation and highlights the withdrawal of FDA support for CSP VITEK2 AFST for *N. glabrata* due to inconsistencies [25, 26]. EUCAST breakpoints have also not yet been established for CSP due to the significant variation in MIC ranges which impedes the calculation of epidemiological cutoff values. Nonetheless, it is inferred that isolates displaying susceptibility to either AFG or MYC are also susceptible to CSP [17].

Espinel-Ingroff *et al*. first explored this concern regarding the variability of CSP MICs obtained via the CLSI BMD [22]. On review of 2750 isolates of *N. glabrata* from 17 different laboratories in Brazil, Canada, Europe, Mexico, Peru and the USA, it was concluded that the use of the CLSI species-specific CSP clinical breakpoints leads to an excessive number of wild-type (WT) isolates being reported as either non-WT or resistant due to the wide variation in MICs generated. However, minimal variation was observed with AFG, suggesting that the issue is CSP-specific and not a general echinocandin issue [22].

Fraser *et al*. followed with a review of echinocandin resistance among *N. glabrata* submitted to the UK National Mycology Reference Laboratory, Public Health England, Bristol, from 2003 to 2016 [21]. Within the first decade of AFST, CSP was assessed against 7225 clinical isolates of *N. glabrata* from seven participant European laboratories. Resistance rates ranged from 0.3 to 7.9 % using the then CLSI breakpoints. However, when current breakpoints were applied to this dataset, the vast majority of isolates were classified as non-susceptible, reinforcing the phenomenon that CSP AFST by BMD – whether manual or automated – artificially inflates resistance rate. With the introduction of AFG as a sentinel echinocandin and specific E-tests for CSP AFST, the resistance rate was actually found to be 0.9–2.7 % [21].

AFST of *N. glabrata* is done locally, with the same isolates also dispatched to the MRL for confirmatory MICs for selected antifungals, namely CSP. At the MRL, AFG acts as their sentinel echinocandin for detecting class-wide resistance and MICs by CLSI broth microdilution are reported as ‘echinocandin’ susceptibility and not agent-specific. When CSP MICs are requested specifically, as in our situation, E-tests are used, which have been shown to be consistent [21, 27].

In addition, while MYC is not used as a therapeutic agent at our hospital, it has been reported to perform more reliably than CSP during AFST with both manual and automated BMD and E-tests. Lim *et al*. reported that AST-YS08 CSP testing appeared unreliable for *N. glabrata* but AST-YS08 MYC was more dependable [33]. More than 80 % of *N*. *glabrata* isolates labelled as intermediate or resistant to CSP were susceptible to MYC using AST-YS08. Therefore, MYC AFST may serve as an alternative testing surrogate for echinocandin class-wide susceptibility.

As shown by the large proportion of MIEs and MEs, with a CA of only 19.5 %, it can be safely concluded that local testing with the VITEK2 over-estimates resistance, a sentiment shared by the above-mentioned reviews. Subsequently, broader acting antifungals may be prescribed whilst awaiting confirmatory MICs, resulting in increased costs and risks of adverse events. Yet, no VMEs were made that could have had detrimental impacts on patient outcomes.

We do recognize the limitations of our study. Being performed at a single-centre in a defined geographical region with a small sample size of isolates is a significant limiting factor in drawing impactful conclusions. Inclusion of other institutions with additional isolates can serve to strengthen the reported findings and represents an area of future work. CA between CSP and MYC using VITEK2 AST-YS08 and results from the MRL also represents an avenue of further evaluation to assess the reliability of MYC as a possible sentinel echinocandin against *N. glabrata*. CSP E-tests may also be considered to overcome uncertainties with CSP VITEK2 AFST but would require further validation and a cost-analysis.

## Conclusion

The detection of antifungal resistance through reliable AFST is paramount toward achieving effective patient care. While the VITEK2 system is a fast and highly applicable semi-automated system, its performance for CSP AFST for *N. glabrata* leaves much to be desired – with an over-estimation of resistance – as echoed by previous studies. As CLSI BMD methodologies are impractical for the routine clinical laboratory, the use of VITEK2 AST-YS08 MYC as a sentinel echinocandin should be explored and/or the evaluation of CSP-specific E-tests as utilized by the MRL. These methods appear more consistent and less prone to the inter-laboratory variation as seen with BMD.
